# Evaluation of an Interdisciplinary Educational Program to Foster Learning Health Systems: Education Evaluation

**DOI:** 10.2196/54152

**Published:** 2025-01-14

**Authors:** Sathana Dushyanthen, Nadia Izzati Zamri, Wendy Chapman, Daniel Capurro, Kayley Lyons

**Affiliations:** 1 Centre for Digital Transformation of Health, University of Melbourne, Carlton, Australia; 2 Faculty of Pharmacy and Pharmaceutical Sciences, Monash University, Parkville, Australia; 3 School of Computing and Information Systems, University of Melbourne, Melbourne, Australia

**Keywords:** continuing professional development, learning health system, flipped classroom, digital health informatics, data science, health professions education, interdisciplinary education, foster, foster learning, health data, design, innovative, innovative solution, health care workforce, Australia, real time, teaching model

## Abstract

**Background:**

Learning health systems (LHS) have the potential to use health data in real time through rapid and continuous cycles of data interrogation, implementing insights to practice, feedback, and practice change. However, there is a lack of an appropriately skilled interprofessional informatics workforce that can leverage knowledge to design innovative solutions. Therefore, there is a need to develop tailored professional development training in digital health, to foster skilled interprofessional learning communities in the health care workforce in Australia.

**Objective:**

This study aimed to explore participants’ experiences and perspectives of participating in an interprofessional education program over 13 weeks. The evaluation also aimed to assess the benefits, barriers, and opportunities for improvements and identify future applications of the course materials.

**Methods:**

We developed a wholly online short course open to interdisciplinary professionals working in digital health in the health care sector. In a flipped classroom model, participants (n=400) undertook 2 hours of preclass learning online and then attended 2.5 hours of live synchronous learning in interactive weekly Zoom workshops for 13 weeks. Throughout the course, they collaborated in small, simulated learning communities (n=5 to 8), engaging in various activities and problem-solving exercises, contributing their unique perspectives and diverse expertise. The course covered a number of topics including background on LHS, establishing learning communities, the design thinking process, data preparation and machine learning analysis, process modeling, clinical decision support, remote patient monitoring, evaluation, implementation, and digital transformation. To evaluate the purpose of the program, we undertook a mixed methods evaluation consisting of pre- and postsurveys rating scales for usefulness, engagement, value, and applicability for various aspects of the course. Participants also completed identical measures of self-efficacy before and after (n=200), with scales mapped to specific skills and tasks that should have been achievable following each of the topics covered. Further, they undertook voluntary weekly surveys to provide feedback on which aspects to continue and recommendations for improvements, via free-text responses.

**Results:**

From the evaluation, it was evident that participants found the teaching model engaging, useful, valuable, and applicable to their work. In the self-efficacy component, we observed a significant increase (*P*<.001) in perceived confidence for all topics, when comparing pre- and postcourse ratings. Overall, it was evident that the program gave participants a framework to organize their knowledge and a common understanding and shared language to converse with other disciplines, changed the way they perceived their role and the possibilities of data and technologies, and provided a toolkit through the LHS framework that they could apply in their workplaces.

**Conclusions:**

We present a program to educate the health workforce on integrating the LHS model into standard practice. Interprofessional collaborative learning was a major component of the value of the program. This evaluation shed light on the multifaceted challenges and expectations of individuals embarking on a digital health program. Understanding the barriers and facilitators of the audience is crucial for creating an inclusive and supportive learning environment. Addressing these challenges will not only enhance participant engagement but also contribute to the overall success of the program and, by extension, the broader integration of digital health solutions into health care practice and, ultimately, patient outcomes.

## Introduction

As health care delivery evolves in complexity and scope, the need for systems that promote continuous learning and adaptation is paramount. The learning health systems (LHS) concept has emerged as a transformative framework that bridges clinical practice with ongoing research, ensuring that health care institutions remain at the forefront of scientific and patient-centered care advancements [1,2]. Central to the LHS paradigm is the notion that data contribute to a broader system of knowledge and is used to refine care practices in real time [1,3]. Achieving this idea requires an interdisciplinary workforce adept in information systems, informatics, data interrogation, quality improvement and implementation methods, and system-based practice, to be able to use the existing data to inform future care [3]. Moreover, health care transformation such as this requires the skills of various professions working together towards solving these complex problems [4,5].

While there are previous studies that have described their LHS-focused programs, few have robustly evaluated the purpose of their implementations. Furthermore, other programs have focused on specific cohorts of participants such as PhD students [6], postdoctoral students [7,8], and clinical fellows [9-11] in the United States [6,9-11] and Canada [7]. Our study adds new insights to the literature given the interprofessional nature of the program, as well as its design (flipped classroom, working groups) and delivery (wholly online). To our knowledge, few programs have involved teaching a structured curriculum [8,12], while other programs have involved mainly project-based work and on-the job learning [7,10,13,14].

For such a dynamic and integrated approach to take root, educating the next generation of health care professionals about LHS principles is crucial. While the theoretical foundation of LHS has been well established, there has been a paucity of research evaluating the efficacy and impact of educational interventions centered on LHS. We developed a 13-week short course called Applied Learning Health Systems, which commenced in September 2021 and has now been running for 2 years [15]. The program is open to all professionals working in the health care setting—clinical and nonclinical—and focuses on interdisciplinary work; the LHS concept can be taught to both digital health and informatics generalists and specialists, clinicians and nonclinicians, front-line workers, and upper management [15].

As institutions increasingly incorporate LHS into their curricula, understanding the nuances of its educational translation becomes vital. This research aims to evaluate the motivations, experiences, and perceptions of participants learning in a collaborative learning environment, as well as the effectiveness, confidence, applicability, challenges, and outcomes of LHS education, providing insights that will shape pedagogical strategies and potentially influence the future of health care education.

The purpose of this paper is to explore participants’ experiences and perspectives of participating in a wholly online interprofessional education program. This evaluation also aimed to assess the benefits, barriers, and opportunities for improvements, and identify future applications of the course materials to the participants’ workplace endeavors. We will also discuss the implementation, feasibility, and outcomes of the program which aimed to foster LHS skills in the Australian health care workforce through didactic coursework, interactive workshops, and collaborative learning. By describing our program and its 2-year evaluation, we believe that current and future educators can learn from our experience when building their own programs. Additionally, our paper will contribute to the emerging education literature on how to foster LHS through workforce development and education. Compared to previous publications on LHS education programs, we are contributing novel insights to this literature through new perspectives based on our location (ie, Australia), the health system data infrastructure (ie, recent electronic medical record [EMR] implementations and digital immaturity), and our participants (ie, diverse interprofessionals). While we have had early successes, we also wish to highlight the obstacles we encountered and how we refined our approach in response. Our results will be valuable to other educators as they consider similar endeavors.

## Methods

### Study Design and Recruitment

We undertook a mixed methods study consisting of both quantitative and qualitative data collection methods. Surveys were conducted precourse, throughout teaching, and postcourse. The surveys consisted of metric scales, qualitative scales, and open free-text boxes. Participation in the research project was via opt-out. Therefore, all enrolled participants were eligible to participate in the project voluntarily, unless they chose not to. There were several modes of recruitment for the course itself. These included reaching out to existing precinct partners who undertook internal expression of interest recruitment processes to sponsor a number of places, social media advertising on X and LinkedIn, Google search search engine optimization, and university students undertaking electives or formal university-accredited certificates.

### Ethical Considerations

This study was approved by the University of Melbourne ethics committee (project ID 22641). In certain parts of the study, participants had the option to opt out (eg, surveys) or provide consent to participate (eg, interviews). In terms of informed consent, participants were provided with a plain language statement describing the purpose and design of the study. Participants were notified that participation was voluntary and were given the option to opt out. For privacy and confidentiality, data were completely deidentified and only aggregate data were analyzed and presented. Data were housed on secure University of Melbourne single sign-on Qualtrics servers and restricted access to OneDrive servers. As participation was completely voluntary, no compensation was provided to participants; however, participants in the pilot version of the course were given free scholarship admission in return for their feedback.

### The Program

The LHS short course was created by the University of Melbourne Centre for Digital Transformation of Health, a high-research academic institution with existing partnerships with local and regional hospitals and primary care networks. The course has been delivered 5 times to 400 participants. Each iteration of the short course involved a 13-week online course revolving around LHS and was delivered wholly online, by diverse instructors, in a flipped classroom learning format. Participants were from a range of backgrounds, including working professionals in health care, PhD research students, masters-level university students, and consumers. The course structure involves 3 hours of weekly individual asynchronous prereadings, followed by 2.5 hours of weekly workshops. Each week participants work through activities associated with a threaded diabetes case scenario, in their assigned interprofessional working group [15].

**Figure 1.**
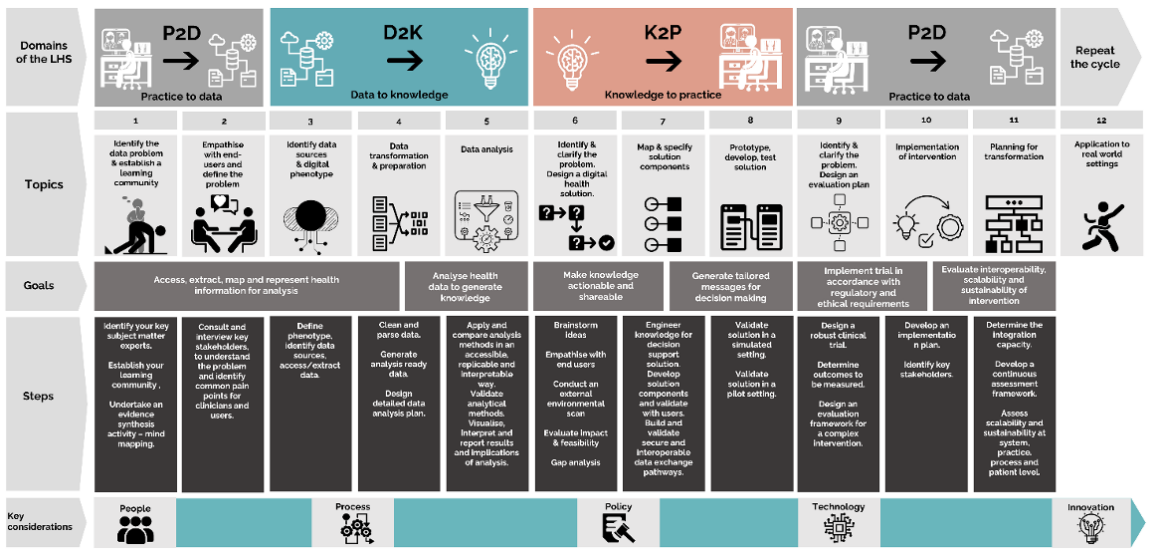
The Applied Learning Health Systems short course curriculum [16].

We mapped the stages of the LHS system onto a swim lane diagram and created specific learning objectives for skills and knowledge at each stage, which were then operationalized into the diabetes scenario. Filling in this swim lane and competency map required knowledge from many disciplines, including data science and biostatistics, standards, user-centered design, change management, workflow mapping, app development, implementation science, and evaluation as well as expertise in the clinical domain and in how the Australian health system works. No single person could effectively design the course we developed, which posed challenges and opportunities for curriculum development. Using the LHS cycle enabled curriculum designers to join the varied subject matter expertise, by mapping it to an agreed framework. Details of the full course design, development, and curriculum outline are published elsewhere (Figure 1) [16].

### Evaluation Framework

We used the Kirkpatrick model of evaluation [17] to map out our measurements (Table 1). This model is a widely used evaluation framework in education and is used to shift researchers away from simply measuring perceptions and satisfaction. We examined whether participants’ attitudes, knowledge, behavior, and professional practice changed as a result. Additionally, we applied a mixed methods approach that included pre- and postsurveys, weekly surveys, and postinterviews.

**Table 1.** Application of the Kirkpatrick model of evaluation, adapted from Barr et al [18], to this project.

Level	Details	Evaluation measures and data sources in this project
1	Perception of training among subjects	Pre-, weekly, and postsurveys; postcourse participant interviews
2a	Change in the attitudes of subjects	Pre- and postchange in digital health interest and identity
2b	Change in the knowledge and/or skills of subjects	Pre- and post-self-efficacy changes in specific LHS^a^ concepts (skills); pre- and postconcept maps (knowledge; out of scope for this paper)
3	Changes in the behavior of subjects	Postcourse participant interviews (will follow up in 1 year with participant interviews)
4a	Change in professional practice	Postcourse participant interviews (will follow up in 1 year with participant interviews)
4b	Changes in patients’ condition	Not applicable

a LHS: learning health system.

### Pre- and Postcourse Surveys

The pre- and postcourse surveys were developed by using a combination of psychological scales and open-ended questions. The pre- and postcourse surveys included the same self-efficacy scale (100 points; cannot do at all to highly certain can do) [19] which has significant evidence of reliability and validity. We choose to evaluate self-efficacy as it is one of the strongest proxy measures in education to predict actual and future performance, which are more difficult and take longer to measure [20]. The 10 items on the self-efficacy scale were adapted from the material taught in the LHS course and language from the LHS literature (eg, use machine learning algorithms to create a model for predicting a health outcome) [21,22]. The open-ended questions included demographic questions (eg, job title) and questions related to digital health identity development, course benefits, course barriers, what to keep, what to improve, and other suggestions or comments.

Surveys were designed and distributed via Qualtrics. Participants were invited to complete the surveys through emails and the learning management system. Responses to open-ended survey questions were also analyzed through qualitative content analysis. Two coders independently coded the text responses using NVivo (Lumivero) software. Coders met to resolve discrepancies and solidify themes and categories under each research question. The self-efficacy scales were analyzed using a 2-tailed, unpaired *t* test in GraphPad Prism to determine whether there was an improvement in self-efficacy across the 13 LHS concepts.

### Weekly Surveys

Over the 12 weeks, participants had the opportunity to provide feedback on the level of engagement, usefulness, value, satisfaction, and areas for improvement in the course content, through participation in weekly surveys. These surveys contained scales (strongly disagree to strongly agree) and ask questions such as “how useful did you find this topic” and “how engaged did you feel” and open boxes for free-text responses. Descriptive statistics such as frequency, mean, and standard deviation will be used to summarize the data from these questions. Completion of these weekly surveys ranged from 2530 participants each week.

### Qualitative Coding of Free-Text Responses

To analyze the text response according to our research questions, we first deidentify the transcripts for participant and institution names. The transcripts will be uploaded to NVivo software for qualitative content analysis [23]. A codebook was developed deductively from the literature and inductively from the research data. Two coders independently analyzed the transcripts according to the codebook. The 2 coders met to calculate an interrater agreement rate and resolve any discrepancies. The final codes were synthesized by creating summaries, narratives, and matrices. The final results included coding frequencies, themes, and categories according to the research questions.

### Quantitative Statistical Analysis

For descriptive statistics, number of participants and proportion of participants are shown. For rating scales, frequency and proportion are shown. Pre- and postcourse self-efficacy comparisons were undertaken using a 2-tailed, unpaired *t* test. Incomplete or missing data were excluded from the analysis.

## Results

### Demographics

Thus far, the Applied Learning Health Systems program has had approximately 400 participants from various organizations (health care, government, research or university, industry) and job roles (clinician, researcher, data or information technology [IT], health services management, allied health, EMR implementation, health administration, consumer advocacy) (Table 2). Of the 400 participants, 343 (85.8%) completed the presurvey (week 0) and 200 (50%) completed the postsurvey (week 12). A few participants were lost to follow-up during the final week because they were ill, dropped out due to overcommitment, or did not respond to requests.

**Table 2.** Demographics shared by participants in the Applied Learning Health Systems program.

Characteristic		Participants, n (%)
**Professional background (n=399)**		
	Primary health care	44 (11)
	Tertiary health care	141 (35.3)
	Health services management	29 (7.3)
	Allied health	48 (12)
	Government	10 (2.5)
	Academia or research	73 (18.3)
	Business, IT^a^, tech or data analytics	47 (11.8)
	Other	7 (1.8)
**Role type (n=343)**		
	Clinician (medical)	67 (17)
	Clinician (nursing)	25 (6.4)
	Clinical informatician	22 (5.6)
	Researcher (health services research or public health)	68 (17.3)
	Data analyst	28 (7.1)
	Allied health professional	58 (14.8)
	Health services manager	36 (9.2)
	Quality improvement lead	24 (6.1)
	Consultant or IT professional	19 (4.8)
	EMR^b^ implementation team	18 (4.6)
	Health administration	8 (2)
	Consumer advocate	20 (5.1)

a IT: information technology.

b EMR: electronic medical record.

### What Were Participants’ Previous Encounters With the LHS Framework?

At the beginning of the course, participants were asked if they had any previous exposure to the LHS framework. Almost one-third of the participants had no previous experience with the LHS concept or any digital health concepts (121/343, 35.3%). Some participants stated that they had previous exposure to digital health and informatics concepts (50/343, 14.6%) through other courses and certifications (27/343, 7.8%), as well as through work-based activities, for example, EMR implementation and optimization (47/343, 13.1%), quality improvement, data interrogation (56/343, 16.3%), and various other health services projects (45/343, 13.1%). Others stated that they had no previous exposure to digital health or LHS concepts (49/343, 14.2%).

### What Type of Teaching Approaches Did Participants Perceive as Effective?

Participants were asked to rate the usefulness and engagement of the topic’s preclass learning and in-class sessions. In terms of usefulness, the majority found the preclass materials useful (880/956, 92.1%*—*“the preclass material was excellent and really helped to clarify many of the terms that I had heard people say but not truly understood”) and in-class sessions useful (902/955, 94.5%*—*“analyzing the data during the class was useful and to see it connect with prelearning materials was good”). When asked to rate engagement, the majority found the preclass (881/954, 92.3%) and in-class activities engaging (881/955, 92.3%) (Tables 3-6).

**Table 3.** Ratings of usefulness and engagement with preclass learning materials and in-class Zoom sessions. Participants were asked to rate the agreement for usefulness (extremely useless to extremely useful) and engagement (extremely unengaged to engaged), weekly for each topic (1-13).

Questions	Rating							Total, n
	Extremely useless, n (%)	Moderately useless, n (%)	Slightly useless, n (%)	Neither useful nor useless, n (%)	Slightly useful, n (%)	Moderately useful, n (%)	Extremely useful, n (%)	
I found this topic’s pre-class learning useful (13 topics)	5 (0.5)	39 (4.1)	13 (1.4)	20 (2.1)	210 (22.0)	265 (27.7)	404 (42.3)	956
I found this topic’s in-class session useful (13 topics)	2 (0.2)	13 (1.4)	9 (0.9)	28 (2.9)	103 (10.8)	360 (37.7)	440 (46.0)	955
I felt engaged when completing the pre-class learning for this topic (13 topics)	5 (0.5)	9 (0.9)	29 (3.0)	30 (3.1)	127 (13.3)	428 (44.9)	326 (34.2)	954
I felt engaged when participating in the topic’s in-class session (13 topics)	10 (1.0)	13 (1.4)	17 (1.8)	33 (3.5)	104 (10.9)	349 (36.5)	429 (44.9)	955

**Table 4.** Participants’ ratings of value pertaining to overall value to personal career development for all topics.

Question	Rating					Total, n
	Highly unvaluable, n (%)	Unvaluable, n (%)	Neutral, n (%)	Valuable, n (%)	Highly valuable, n (%)	
Valuable to your personal career development (13 topics, n=189)	12 (0.6)	13 (0.6)	251 (12.3)	989 (48.5)	776 (38.0)	2041

**Table 5.** Participants’ ratings of value pertaining to applicability to current workplace role for all topics.

Question	Rating					Total, n
	Highly not applicable, n (%)	Not applicable, n (%)	Neutral, n (%)	Applicable, n (%)	Highly applicable, n (%)	
Applicability to your current workplace role (13 topics, n=189)	64 (3.1)	154 (7.5)	325 (15.9)	837 (41.0)	661 (32.4)	2041

**Table 6.** Participants’ ratings of value pertaining to overall satisfaction with the quality of the course, recommendation, instructors, and choice to revisit, as well as the value of educational activities (instructors, Zoom workshops, Canvas preclass activities, collaborative learning, the diabetes case scenario, Jupyter Notebooks, and discussion boards).

Questions	Rating							Total, n
	Extremely valueless, n (%)	Moderately valueless, n (%)	Slightly valueless, n (%)	Neither valuable nor valueless, n (%)	Slightly valuable, n (%)	Moderately valuable, n (%)	Extremely valuable, n (%)	
Collaborative learning in the working groups	1 (0.5)	6 (3.3)	3 (1.6)	5 (2.7)	29 (15.9)	63 (34.6)	75 (41.2)	182
Preclass learning activities on Canvas	0 (0)	3 (1.6)	2 (1.1)	2 (1.1)	21 (11.5)	77 (42.3)	77 (42.3)	182
In-class learning (Zoom) sessions	1 (0.5)	2 (1.1)	1 (0.5)	6 (3.3)	17 (9.3)	74 (40.7)	81 (44.5)	182
The diabetes case scenario	1 (0.5)	4 (2.2)	3 (1.6)	10 (5.5)	30 (16.5)	70 (38.5)	64 (35.2)	182
Jupyter Notebooks	0 (0)	11 (6.0)	11 (6.0)	20 (11.0)	53 (29.1)	55 (30.2)	32 (17.6)	182
Canvas learning management system	0 (0)	1 (0.5)	3 (1.6)	12 (6.6)	30 (16.5)	83 (45.6)	53 (29.1)	182
Discussion boards	5 (2.7)	10 (5.5)	10 (5.5)	43 (23.6)	62 (34.1)	39 (21.4)	13 (7.1)	182
The instructors	0 (0)	1 (0.5)	0 (0)	4 (2.2)	8 (4.4)	49 (26.9)	120 (65.9)	182

Responses to the question of satisfaction also yielded highly positive results. For the overall quality of the short course, most agreed that it was of a high standard (178/182, 97.8%), including the instructor quality (175 /182, 96.2%). When asked if they would recommend the short course to a colleague, 89.5% (163/182) said they would. In terms of revisiting the decision to complete it again, 85.1% (154/182) still said they would choose to take the course. When rating the value of the course to their personal career development, a majority found the course valuable (173/200, 86.5%). Participants were also asked to rate the applicability of the course to their day-to-day work, where 73.4% (134/182) found it applicable.

Given the number of facets implemented in the course, we asked participants to rate the value of these various elements. The most highly rated was the instructors: “the speakers were great, and the structure of having a short lecture and then doing an activity then coming back and having another lecture was good,” with 92.8% (169/182) finding them moderately or extremely valuable. Next, in-class learning (155/182, 85.2%), preclass learning (154/182, 84.6%), collaborative learning (138/182, 75.8%), the diabetes case scenario (134/182, 73.7%), and the Canvas learning management system platform (136/182, 74.7%) rated similarly. The use of Jupyter Notebooks (87/182, 47.8%), and the discussion boards (52/182, 28.6%) rated lower (Table 6).

### How Did Participants’ Self-Efficacy for Digital Health Topics Change After the Course?

**Figure 2.**
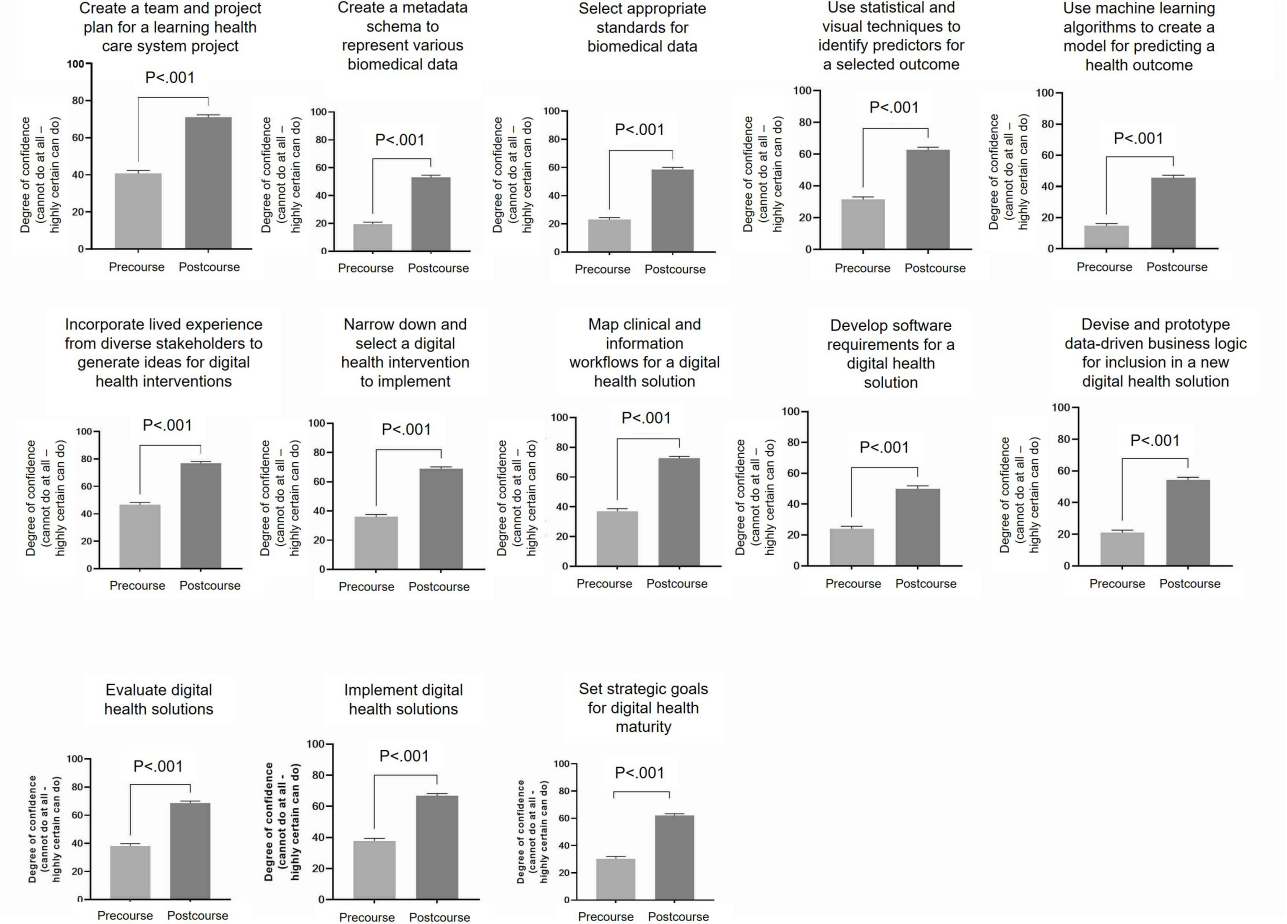
Pre- and postcourse self-efficacy in LHS concepts. Participants rated confidence on a scale of 0‐100 (0=cannot do at all to 100=highly certain can do). Two-tailed, unpaired *t* test was undertaken (n=296 precourse, n=200 postcourse). Changes from baseline to postcourse confidence are shown for each LHS concept. LHS: learning health system.

To explore the change in self-confidence levels pre- and postcourse, participants were surveyed on the key competencies for the 13 topics. Participants completed the same set of ratings at the beginning and at the end of the course, following completion of all the material. For all 13 learning outcomes, there was a statistically significant increase in self-efficacy (n=200, *P*<.001) (Figure 2).

### How Did Participants’ Self-Perceived Role in Digital Health Change?

In the pre- and postsurvey, participants were asked to respond to the open-ended question of “What do you see as your role in digital health?” There were several types of roles that participants perceived themselves embodying postcourse. These included users of digital health or learners; champions, advocates, or change agents; researchers, innovators, or entrepreneurs; leaders, managers, strategic planners, or decision makers; educators or mentors; specialists or implementers; community builders, connectors, facilitators, collaborators, or translators (Table 7).

After the course, there was an increase in participants who viewed their role as an end user or learner and a community builder or facilitator, whereas there was a decrease in those who viewed their role as a champion or advocate and leader in digital health.

**Table 7.** Participants’ perceived roles in digital health pre- and postcourse (qualitative themes).

	Precourse responses (n=274), n (%)	Postcourse responses (n=228), n (%)
End user of digital health or learner	41 (15.0)	54 (23.7)
Champion, advocate, or change agent	62 (22.6)	37 (16.2)
Researcher, innovator, or entrepreneur	32 (11.7)	30 (13.2)
Leader, manager, strategic planner, or decision maker	38 (13.9)	22 (9.6)
Educators or mentors	13 (4.7)	6 (2.6)
Specialist or implementer	57 (20.8)	41 (18.0)
Community builder, connector, facilitator, collaborator, or translator	31 (11.3)	38 (16.7)

### What Did Participants Perceive as the Applications of the Learning in Their Workplace?

There were five main themes that arose for the types of applications that participants foresaw themselves using the course learnings: (1) learning and professional development: “upskilling in the current role, more understanding of the roles of my team members”; (2) using data and undertaking data analysis more effectively: “data mining and improving processes at work”; (3) implementing the LHS framework for digital health interventions: “we are embarking on establishing a data and analytics 3-year plan and we intend to incorporate LHS principals into this strategy”; (4) for undertaking research and quality improvement activities: “I now play a role in learning health networks for Safer Care Victoria, where I believe I could encourage digital health projects focused on quality improvement and patient safety”; and (5) collaborating and sharing knowledge and learnings with colleagues: “I intend to instill the LHS framework into my role, the work that I do and share it with my team” (Table 8).

**Table 8.** Participants’ anticipated applications of learning in the workplace (qualitative themes).

	Precourse responses (n=338), n (%)	Postcourse responses (n=231), n (%)
Learning and professional development	74 (21.9)	47 (20.4)
Using data and undertaking data analysis	54 (16.0)	43 (18.6)
Implementing digital health solutions with the LHS^a^ framework	63 (18.6)	52 (22.5)
Researching and quality improvement	82 (24.3)	62 (26.8)
Collaborating and knowledge sharing	65 (19.2)	27 (11.7)

a LHS: learning health system.

### What Were the Perceived Benefits of the Program?

Participants were asked to state the benefits of the program. The major themes that arose were learning and knowledge acquisition: “the course material was presented well on Canvas and had a good mix of different learning resources to use,” value of collaboration: “the course has been extremely eye-opening and has led me to begin collaborations on digital health projects through contacts made through the course,” participant diversity and group work : “being in a group of people with all different work backgrounds and skills coming together with a common interest was really good for tackling the problems to solve in the class,” beneficial course structure and content delivery (preclass: “the course material was presented well on Canvas and had a good mix of different learning resources to use” and in-class: “beneficial to be in a diverse group of other health care professionals - I learnt a lot from the robust and engaging discussions on Zoom),” and learning tools, importance of real-world applications: case study and personal work: “applying course concepts to this real-world scenario was instrumental in reinforcing their understanding,” appreciation for instructors’ diversity, expertise, engagement, and quality: “the instructors were very engaged and passionate about their topics,” consumer focus, and focus on data analytics (Table 9).

**Table 9.** Beneficial elements of the course.

Theme	Responses (n=295), n (%)
Collaborative group work, diversity, or multidisciplinary approach	63 (21.4)
Course structure and content delivery or pre- and in-class material	54 (18.3)
Learning and knowledge acquisition	53 (18.0)
Real-world scenarios or real-world applicability	44 (14.9)
Exposure to tools and techniques	28 (9.5)
Appreciation for instructors	25 (8.5)
Exposure to complexity and challenges	14 (4.7)
Focus on consumers	14 (4.7)

### What Were Participants’ Barriers to Engaging With the Program?

When asked regarding barriers to participating in the course, participants’ responses formed the following major categories: time constraints due to work, family, and other social commitments: “time constraints, balancing clinical work, other non-clinical work and home life,” a lack of knowledge, terminology, and experience: “limited coal-face/frontline exposure and visibility of emerging frontline issues. I work at a more systems-based level and am not involved in interacting with patients day-to-day,” technical challenges: “I found using so many new platforms eg Jupyter notebooks, BPMN so quickly challenging...,” content complexity, and limited interactions online (Table 10).

**Table 10.** Barriers to effective participation.

Theme	Responses (n=259), n (%)
Time constraints or keeping up with materials	109 (42.1)
Lack of knowledge and experience	56 (21.6)
Family and personal commitments	37 (14.3)
Technical challenges	23 (8.9)
Health care terminology and clinical knowledge	14 (5.4)
Work commitments	14 (5.4)
Course structure and content	6 (2.3)

### What Changes or Improvements Would Participants Suggest to the Short LHS Coursework?

While the majority of participants found beneficial elements to the course, there are always improvements that can be made. Areas in which changes were suggested were course structure, duration, and timing, suggesting concerns around the pace of the course and the amount of information and breadth covered: “it feels like a lot of materials are being cramped into 1 session and it was hard to appreciate the differences between the models” and the timing of delivery after a long work day; the usability of some learning tools, such as Jupyter Notebooks, difficulties with learning management platform navigation, more revision activities to reinforce learning and a desire for more printable or downloadable resources; questionable benefit of group work and collaborative work where students wanted more support and time to hear instructor expertise: “I feel there was too much reliance on group work and not enough input and guidance from the experts”; course delivery—online format, questioning whether networking opportunities were lost online; prerequisite skills required, given the difficulty of some content (Table 11).

**Table 11.** Participants suggested improvements to the course.

Theme	Responses (n=158), n (%)
Course content and structure—curriculum, quality, volume of material, level of complexity, clarity, usefulness, effectiveness, engagement, and applicability	64 (40.5)
Course logistics and administration—course duration, pace, delivery modality, pre-requisites, and learning platforms	37 (23.4)
Learning tools and materials—usability and accessibility	15 (9.5)
Group work and collaboration activities—diversity, effectiveness, and interaction	30 (19.0)
Instructor interactions in-class—interaction, engagement, and support	12 (7.6)

## Discussion

### Principal Findings

Despite the concept originating in 2007 [24], there is a lack of reports evaluating LHS education programs. In this evaluation, we discuss the findings of 2 years of implementation and iteration of an interdisciplinary Applied LHS professional development course (343/400, 85.8%, presurvey respondents; 200/400, 50%, postsurvey respondents), to a diverse range of professionals working and studying in health care, with an interest in digital health. Most of our participants were from Australia, where LHS was a novel but emerging concept [15,25-27]. The participants found the course engaging and relevant to their work. Participants highlighted specific benefits, barriers, and applications to this course and the LHS framework on their work.

Most health systems are actively seeking to increase the use of data and digital technology to drive improved health care delivery and health outcomes. A major ingredient needed to achieve that lofty goal is a workforce that knows how to not only thrive within the rapidly digitizing world but also how to innovate to improve value-driven care. Training a diverse workforce in the digital transformation of health poses an overwhelming number of choices about the most important learning objectives, competencies, and skills. The LHS framework [16] placed boundaries around the grand vision and enabled us to concretely tell a story that resonated with the goals of potential learners while lending itself to hands-on activities that invite learners to be part of that story.

In addition to the advantages of multidisciplinary curriculum development, the LHS framework was also a key part of the value of the course to interdisciplinary learners. We launched this course as a pilot and hand-selected 50 participants from a much larger pool of applicants with the aim of multidisciplinary involvement and of creating buzz around the course to encourage enrolment for a fee-paying version of the course. Medical directors, research leads, clinicians, and managers brought learnings from the course to hallway discussions and team meetings in their workplaces about how they could apply the LHS framework in specific projects. In addition to a better understanding of how a project could go from idea to implementation and evaluation using the LHS principles, the framework provided a shared lexicon, a set of approaches like the creation of a learning community, and a toolkit of methods that learners could envision being used in their work. Their excitement was contagious, and a large proportion of our enrolees have come from organizations who continue to sponsor entire interdisciplinary teams of people to take the course together, because they see the value of the framework as a connector across disparate teams, such as clinicians, IT or EMR analysts, and health intelligence units, seeking to work toward a shared goal.

Overall, the course attracted a wide range of professionals at different levels (eg, medical students to directors of emergency departments), professions (eg, nursing and social work), consumers, researchers, and disciplines (eg, IT professionals). In this study, participants highly valued the interdisciplinary nature and collaborative learning activities in the course. Based on previous educational research, we purposefully sorted the groups for a diversity of professions and kept the participants within the same groups for the majority of the course to encourage relationship building. The interdisciplinary aspect of this course was a strength of our education model as it mimics the type of interdisciplinary practice required for complex LHS and digital health initiatives [28].

From several written comments and weekly surveys, we found that different disciplines struggled at different points within the course. For example, people without a research background found the data analysis topic and using Jupyter Notebooks the most challenging aspect of the course, whereas those with a nonclinical background struggled the most with mapping clinical workflows and implementation. Although we used these struggles as teaching moments to demonstrate the need for an interdisciplinary team in LHS, our experience indicates the need to improve our interdisciplinary education model. Previous education researchers and motivational theorists have established that optimal challenge is a key ingredient for engagement and learning [29]. If the material is too easy or too difficult, then learners disengage and, thus, do not learn the material. Many educators have described the challenge of designing a course for optimal challenge among a large cohort of uniprofessional courses [30]. However, our experience is that this challenge is even more dramatic in a one-size-fits-all model in an interdisciplinary course. The content we taught is still appropriate for all audiences, but each person may require more or less self-directed preparatory work as part of the flipped classroom model. Future researchers and educators should investigate how to continue serving an interdisciplinary audience while creating optimal challenges for all participants. For example, in future iterations, we will explore the use of generative artificial intelligence tools to personalize the self-directed online modules for participants’ previous knowledge and professional context.

The participants’ self-described digital health roles before and after the course only went through minor changes. There was a small conversion in participants who started out seeing themselves as leaders and then later described their roles as connectors. This phenomenon may have been due to instructors telling participants about the importance of connector roles within the LHS framework. Another reason for this effect may be the Dunning-Kruger effect [31]. The Dunning-Kruger effect is when individuals with low exposure to a topic often overestimate their abilities due to a lack of metacognitive awareness. As they gain more knowledge, they become more aware of the limitations. Despite the potential for the Dunning-Kruger effect, the lack of significant changes in participant digital health identity was in contrast to a similar evaluation of our parallel LHS education offering—a 1-year LHS fellowship program for clinicians [15]. In the fellowship program, half of the participants began the program by describing their role as champions and leaders, and then, by the middle of the program, all of the participants described their role as champions and leaders. This potential effect may be due to the benefits of the fellowship program; the fellowship is more experiential, project based, and explicitly focused on leadership development. Since self-identities are an important mediator of future performance [32], future educators and researchers should continue to investigate how LHS educational programs influence participants’ self-described roles in the LHS framework and digital health.

### Strengths, Limitations, and Future Directions

Overall, we achieved commendable survey response rates, suggesting a high level of engagement from participants. This study uniquely contributes to the existing literature by evaluating an interdisciplinary LHS education program—a domain previously underexplored. Our comprehensive approach encompassed both pre- and postcourse survey data, leveraging learning theories such as self-efficacy theory and the Kirkpatrick evaluation framework to inform our evaluation. Moreover, our qualitative analysis offers valuable insights into participants’ perceptions, enriching our understanding of their experiences. However, a limitation is our current inability to capture the upper levels of the Kirkpatrick model, specifically how the LHS course may have influenced participants’ workplace behaviors and the subsequent outcomes of those behaviors. In the long term, we aim to evaluate the impact this course and other LHS education offerings have had on individuals and their health organizations’ journeys toward a learning health system and individual’s career progression. We aim to do this by conducting follow-up, in-depth interviews with participants and organizational sponsors and thematically analyzing the changes that have occurred over time.

Achieving an LHS requires a symbiotic partnership between researchers and health services—by bridging theory and real-world application, future innovations emerging from an LHS will be evidence based and clinically relevant. To increase academic-practice collaboration, our LHS educational offerings aim to grow the understanding of LHS principles and skills in our health services partners and to provide insight into the enablers and barriers for their digital transformation. The shared LHS framework and increased mutual understanding from these programs are increasing trust and collaborative opportunities, leading toward joint translational LHS innovation programs within the health services. We hope that future educators and academic leaders see promise in our emerging LHS education evaluation work [15], other descriptions of LHS education initiatives [6-11], and the success of LHS initiatives in health care practice [33-35].

By providing a professional development short course, we were able to serve a large market of health professionals who would not otherwise have participated in an expensive university degree. While some professionals like medical specialists receive a continuing medical education fund, most other disciplines are not provided with funding for professional development. Additionally, a major source of participants was partner organizations supporting and sending groups of staff through the program, to learn together as cohorts to develop communities of practice. In this scenario, enrollment was funded by their employers. This is crucial, as at the national and international level, we require a critical mass of appropriately skilled workforce to leverage LHS principles in improving the quality and value of health care delivery.

An interdisciplinary LHS short course has also provided a testbed for applying new technologies to learning. For instance, in the last iteration of the course, we experimented with generative AI feedback on the participants’ learning. In their working groups, participants developed an evaluation plan. They fed their plans into ChatGPT, which we provided with structured, custom prompts to provide feedback and rate the quality of the plans. Although some students found the feedback to be generic, the depth of the feedback was dependent upon the richness of the data initially fed to the machine. In large group settings, where there are limited instructors and limited time to provide in-depth feedback to each interdisciplinary group or participant, ChatGPT may be a useful tool to assist with providing formative feedback. The use of this will be further explored in future iterations of the course.

### Conclusions

Overall, the Applied Learning Health Systems course received significant positive feedback from interdisciplinary learners. They found the course to be well structured, engaging, and a valuable learning experience. The qualitative comments emphasized the importance of delivering courses that not only provide knowledge but also inspire and motivate learners, and provide concrete tools to apply in their workplaces. A significant number of participants expressed interest in future courses and opportunities for further learning, underscoring the potential for expanding and diversifying course offerings in the future. There is still a great deal of education that needs to be provided to upskill the workforce adequately enough to undertake digital health transformation, but it begins with a shared vision, a common language, and a mutual framework to follow.

## Abbreviations

EMR: electronic medical record
IT: information technology
LHS: learning health system
